# A community methodological protocol of a multisector collective impact collaboration to address older adult isolation in a rural county in the U.S.

**DOI:** 10.3389/fpubh.2025.1422126

**Published:** 2025-02-12

**Authors:** Christine Marcos, Michael Castellano, Lindsey Skripka, Lenard W. Kaye

**Affiliations:** ^1^Moses Taylor Foundation, Scranton, PA, United States; ^2^United Way of Lackawanna, Wayne & Pike, Scranton, PA, United States; ^3^Meals on Wheels of Northeastern Pennsylvania, Scranton, PA, United States; ^4^Center on Aging and School of Social Work, University of Maine, Bangor, ME, United States

**Keywords:** older adults, isolation, rural, aging, social connection, collaboration, collective impact

## Abstract

In 2019, a community collaborative of nearly 30 health care, social service, philanthropic, and government organizations came together to construct a community-wide plan to reduce older adult isolation in Lackawanna County, Pennsylvania. Although such collaborations have been pursued before, the current one has exceeded expectations, launched a promising pilot, and formed exciting ripple effects throughout the region's aging services landscape. Among the implementation strategies informing the initiative are the use of an interorganizational shared screening tool to identify isolation risk, a team of older adult peer navigators to provide one-on-one assistance and foster connections for those who are isolated, and a major public awareness campaign to educate residents on the negative health impacts of isolation and reduce the stigma felt by many living socially disconnected lives. This article will summarize the methodological process used in developing a cohesive, multi-sector collective impact coalition, as well as examine the limitations and future directions for this initiative.

## 1 Introduction

Social disconnection encompasses both social isolation, defined as an objective lack of social contact with others for extended and measurable periods of time, and loneliness, defined as the subjective experience of social isolation or the personalized and emotional sense of being socially isolated not measured necessarily by the extended passage of time separated from others (1–3). Both social isolation and loneliness present significant public health issues, exacerbated among older adults through biological aging, which is not conducive to maintaining typical social relationships (4). Aging individuals often experience relationship losses, health issues, functional decline, and sensory impairments—factors that can contribute to a lack of social connection (3).

The results of a rigorous meta-analysis study on social isolation and loneliness by the National Academies of Sciences, Engineering, and Medicine in 2018 found that 24% of older adults living independently in communities were considered socially isolated (5). The research also uncovered serious health impacts: social isolation has been associated with an increased risk of mortality, 50% increased risk of developing dementia, 29% increased risk of coronary artery disease, and 32% increased risk of stroke (5). Furthermore, social isolation results in cost to the United States economy estimated at $406 billion annually, and $6.7 billion in additional Medicare spending each year (4). The effects of social isolation are pervasive, impacting the health care system, social services, and the community-at-large.

The Older Adult Social Isolation Collaborative of Lackawanna County (“Collaborative”) aims to mitigate social isolation within older adults living in Lackawanna County utilizing a collective impact model. The purpose of this article is to document the methodological process used for developing and implementing this collective impact model and lay the groundwork for other communities endeavoring to address social isolation collectively.

## 2 Background and rationale

### 2.1 Social isolation

In 2023, the United States Surgeon General, Vivek H. Murthy, released an advisory called “Our Epidemic of Loneliness and Social Isolation,” highlighting some of the impacts of the public health crisis created by lack of social connection (6). This report noted that older adults have been found to have the highest rates of social isolation (6). Addressing older adult isolation in the rural setting is particularly key because while the proportion of individuals 65 years of age and older is increasing throughout the United States, it is increasing more rapidly in rural compared to urban areas (7). A 2019 United States Census Bureau report found that 17.5% of rural populations were aged 65+, compared to 13.8% of urban populations (8). Older adults living in rural communities face a unique risk of social isolation, due to challenges presented by accessibility barriers such as limited transportation, undeveloped infrastructure, and limited available technology (9).

### 2.2 Collective impact model

To address older adult isolation in Lackawanna County, the Collaborative utilized a collective impact model. Collective impact occurs when a group of multi-sectoral stakeholders commit to a common agenda to solve a specific social problem (10). Collective impact models are distinctly different from traditional networks or collaborations in that they involve a centralized infrastructure, dedicated staff, and a structured process that facilitates a common agenda, shared measurement, open communication, and mutually reinforcing activities among the participants (10). The value of this approach lies in the idea that complex social problems require engagement from an array of players from various sectors, including those outside the non-profit realm (10). The use of such a model to address older adult isolation is reinforced by an integrative literature review published in 2021 examining various approaches to enhance social connections at individual and community levels (11). The authors of the review ultimately suggested a comprehensive strategic initiative to be implemented by a collaborative network of community stakeholders, public policymakers, public and private organizations, and the healthcare sector (11). The present case offers a methodological protocol for how such a model can be achieved.

## 3 Context

### 3.1 Lackawanna County

The target population for this initiative is older adults in Lackawanna County, which is a primarily rural county in Pennsylvania. Lackawanna County has a population of 215,615, 20.6% of which consists of individuals 65 years of age and older (12). This is significantly higher than the national average of 17.3% (12). According to an unpublished report on older adult isolation commissioned by Moses Taylor Foundation and produced by The Institute (a local, nonprofit data analysis, research, and consulting organization) when compared to national statistics, Lackawanna County has a higher incidence of nearly all the American Association of Retired Persons (AARP) risk factors for older adult isolation: living alone, having an income below the poverty threshold, being single, and being disabled (13).

In addition to the risk factors defined by AARP, previous scholarship also suggests that opportunities for social engagement decline as health status declines, whether physical or mental (5, 14). The same report from The Institute (privately obtained) found that nearly 28% of adults in Northeastern Pennsylvania age 65 and older reported limited activity due to physical, mental, or emotional problems, indicating significant barriers to maintaining social connections with their informal support networks of family, friends, and neighbors (13). These data, however, merely indicate the prevalence of risk factors for isolation, rather than constituting a direct measure of isolation itself, thus illustrating the difficulty in determining the full extent of isolation within the community. Despite these challenges in direct measurement, the disproportionately large population of older adults in Lackawanna County, combined with the high levels of known risk factors, suggest a need for both targeted and concerted action.

Another important contextual feature is that a significant portion of Lackawanna County is rural, save for the more urban hub of its largest city, Scranton (15). Rural communities have been well-documented as having increased barriers to social connection, such as fewer transportation options and lower walkability ratings, higher poverty levels, decreased access to broadband, and limited health care resources (9).

In addition to substantial rurality, there is significant diversity in some areas of the County, partially because the area serves as a refugee resettlement location (16). For example, in the Scranton School District, there are students who have immigrated from more than 24 different countries (17). Outlying portions of the County tend to evidence less diversity. The overall racial/ethnic make-up is: 81.2% white (not Hispanic or Latino); 10% Hispanic or Latino, 5.1% Black or African American, 3.4% Asian, 2% two or more races, 0.4% American Indian and Alaska Native, and 0.1% Native Hawaiian and Other Pacific Islander (12). Recognizing the diversity in the population is imperative, as the Collaborative aims to serve older adults in need of increased opportunities for social connection in culturally appropriate ways.

### 3.2 The Collaborative

The combination of research related to the severe health impacts of isolation and high levels of local risk factors spurred Moses Taylor Foundation (“Foundation”), a regional health conversion foundation located in Scranton, Pennsylvania, to launch a strategic initiative focused on reducing the negative impact of social isolation on the community's older adults. The groundwork began in 2018, with the Foundation gathering community-wide input on how to address this issue.

Lackawanna County service providers identified numerous existing community programs that older adults could take advantage of to reduce their risk of becoming socially isolated, such as friendly visiting services, specialized exercise programs, and volunteer placement services. However, partners also identified a significant gap in terms of effectively identifying, and subsequently connecting, individuals with those services and programs. In this way, the local aging services system was fragmented, frequently siloed, and in need of stronger collaboration models to best serve the population in need.

A needs assessment conducted by The Institute in 2018 for the Lackawanna County Area Agency (unpublished) on Aging confirmed the disjointed nature of service connection; the older adults surveyed identified lack of information as a key barrier to obtaining needed assistance (18). Informational scarcity is a known key factor in predicting approachability of community services to older residents, as well as a program's level of transparency to the public, the extent of their outreach efforts, and their use of effective screening protocols (9, 19).

With this context in mind, Moses Taylor Foundation designed a Request for Proposals (RFP) that sought a *collaborative* of health and social services providers to work together on first a planning grant, then a pilot grant for an intervention to address older adult isolation in Lackawanna County.

In 2019, the grant was awarded to the lead organization, United Way of Lackawanna, Wayne & Pike (“United Way”), which is seen as a “neutral” organization within the community, eliminating questions of project ownership and allowing a variety of community partners to jointly buy-in to the initiative. The result was a multi-sector group of nearly 30 health care, social service, philanthropic, and government organizations, each with “skin in the game” but none owning the initiative itself.

## 4 Programmatic elements

### 4.1 Current initiative

Upon receiving funding in late 2019, the Collaborative began structuring its approach to addressing older adult isolation utilizing a collective impact model. They had already identified the United Way as their lead organization, but they also hired a national expert to keep them abreast of the latest research around effective interventions to reduce older adult isolation. At the group's monthly full group meetings, and through smaller working committees, they decided on utilization of a tool for common measurement, the Upstream Social Isolation Risk Screener [U-SIRS; (20)], as well as other key programmatic elements (i.e., older adult navigators and awareness campaign).

**Figure 1:**
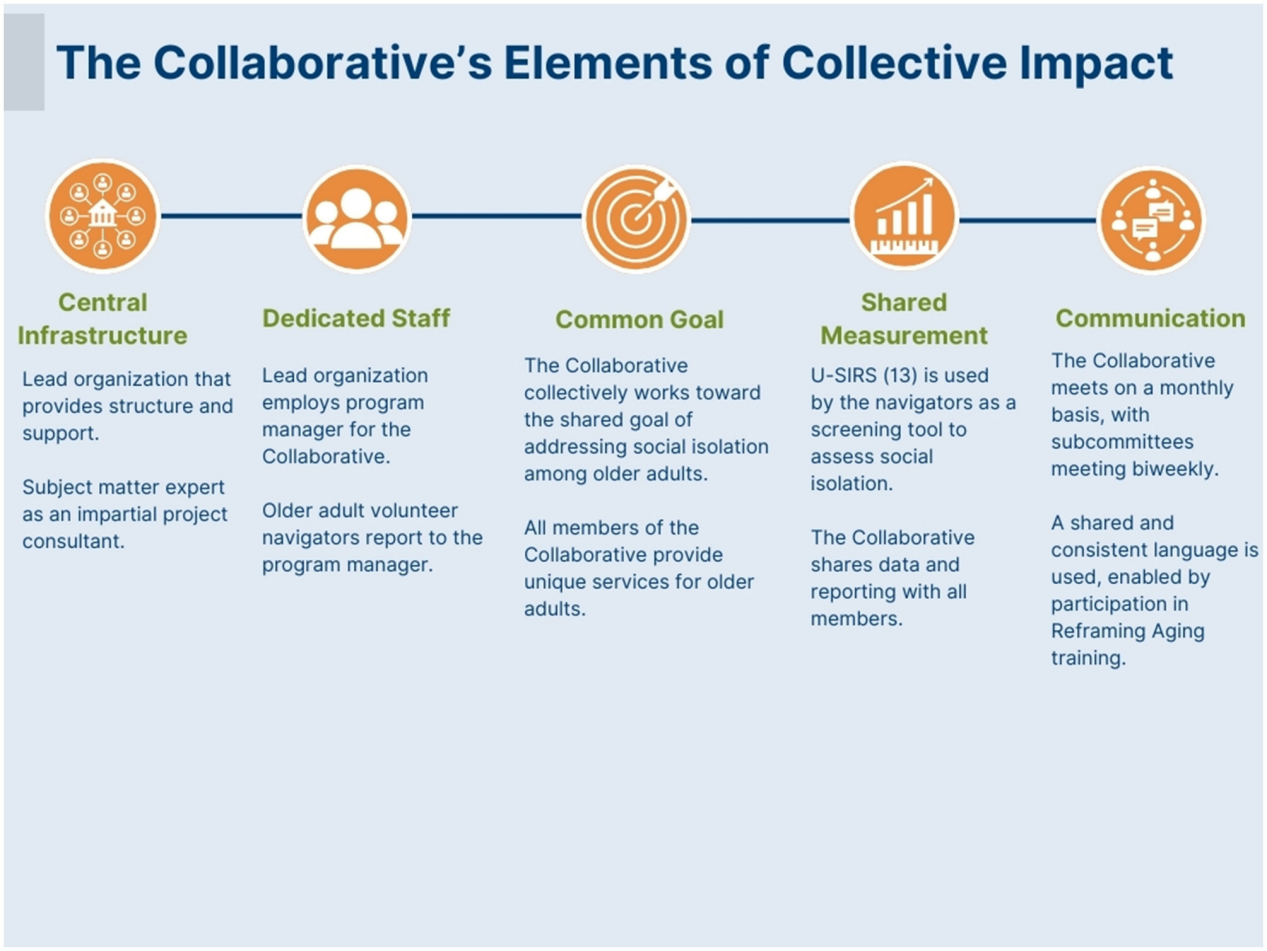
The Collaborative's organizational elements informed by collective impact (10).

When the group transitioned from planning to pilot implementation in 2021, the United Way also hired a dedicated program manager for the Collaborative, which was key to ensuring the group had the facilitative staffing capacity to achieve its common goal. Another component formative to the Collaborative's communication was participation in Reframing Aging training (21) to create a shared and consistent language when conversing about older adult issues, while also being cognizant of avoiding any undercurrents of ageist communication. Figure 1 illustrates further how these key collaborative elements have been informed by the collective impact model as defined by Kania and Kramer (10).

**Figure 2:**
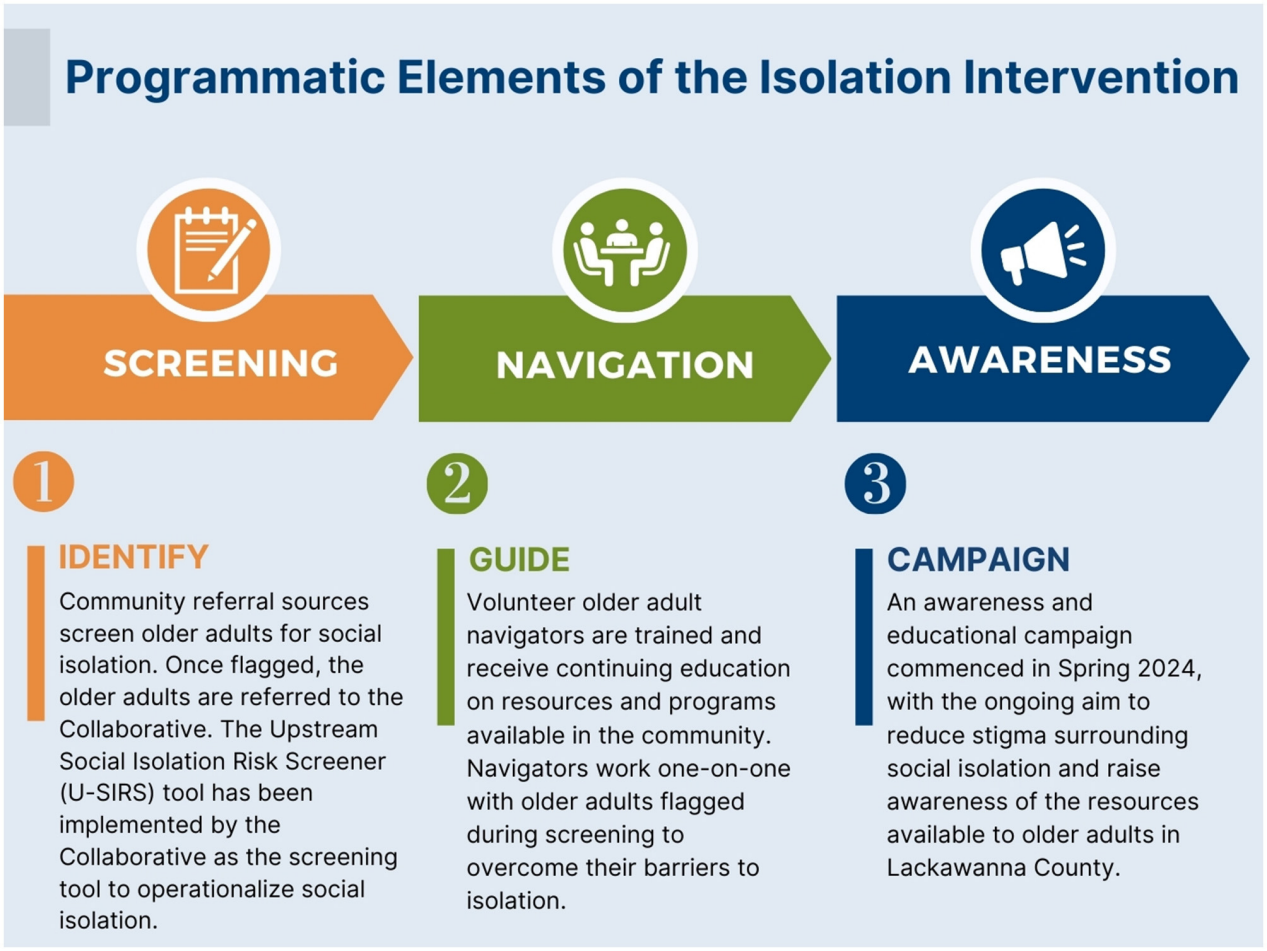
Programmatic elements of the Collaborative's isolation intervention.

With its structure in place, the Collaborative was able to design and begin implementing a three-year pilot (2022-2024) that has three primary programmatic components: Screening, Navigation, and Awareness (Figure 2).

Specifically, a variety of referral sources throughout the community (beginning with health and social service entities) have been engaged to screen older adult patients and clients for loneliness and isolation. Those that are flagged during these screenings are referred to the United Way, where a team of volunteer older adult navigators have been trained to work one-on-one with them to overcome individual barriers to social connection. The third component, Awareness, reinforces the first two components through an ongoing public awareness campaign to reduce stigma and educate the larger community. All components center around formalized protocols adhered to by multiple organizations spanning several sectors in the community and were developed with influence from the theoretical models described in Table 1.

**Table 1:** Evidence-based contributions to the programmatic elements of the Collaborative's isolation intervention (22–25).

**Framework**	**Description**	**Application in programmatic elements**
Social prescribing model	Social connection is considered a lifestyle intervention that is prescribed by physicians for patients who lack social connections. The model includes linkages to related services facilitated by the physician's staff (22)	Conceptual basis for the referral of older adults at risk for social isolation to the Collaborative by health care partners
Gatekeeper services	These services utilize non-traditional referral sources to connect with individuals who are particularly hard to reach. Such models have demonstrated positive results in terms of being able to engage individuals who otherwise would likely never, or only with significant delay, proceed to access and utilize existing community services (22)	Foundation for developing partnerships with a wide variety of referral sources to screen for isolation and loneliness
Levesque, Harris, and Russell “Access to Healthcare Framework”	According to the model, there are five dimensions of barriers to access care: approachability, acceptability, availability/accommodation, affordability, and appropriateness (23)	The Collaborative took all five dimensions of barriers to access into account when identifying methods of identification, screening, navigation, and coordination of services to older adult participants (23)
Systems Of Cross-sector Integration and Action across the Lifespan (SOCIAL) framework	The SOCIAL framework reflects a hybrid relationship of the socio-ecological model and the Health in All Policy (HiAP) framework to illustrate how multiple sectors of society and level of influence can contribute to social connection and reduce social isolation and loneliness (24)	Foundation for the collaborative network of community organizations
Community-based champions, implemented in the program Nav-CARE	Pesut et al. implemented a volunteer-navigation program for older persons with advanced chronic illness called Nav-CARE in Canada. That program emphasized a compassionate community approach to de-medicalize the potentially sensitive and stigmatizing dimensions of palliative care (25)	Foundation for the role of the older adult navigators to be volunteer-based

### 4.2 Activities undertaken

During its 6-month planning phase, the Collaborative developed three subcommittees, one for each of the primary program components. Each subcommittee was tasked with gathering information and making recommendations to the full group for the design of its respective component.

The subcommittee focused on Screening began by researching existing loneliness and isolation screening tools with help from The Institute. U-SIRS was ultimately chosen as the central assessment tool to be used by the navigators.

U-SIRS was developed by Smith and Barrett at Texas A&M University and has been standardized and validated (20). It is a 13-item assessment that measures social isolation risk among community-dwelling older adults and subsequently recommends needed services and programs (20). The primary reason the Collaborative chose U-SIRS was because the suggestion of unmet service needs provides a useful starting point for the navigators to begin their intervention.

Although the Collaborative decided to use U-SIRS as its central screening tool, the Screening subcommittee learned during the process of its work that several health and social service organizations in the community were already required to administer other social isolation and loneliness screenings by their own governing bodies. Recognizing that convincing all entities to change their screening protocols would be a long, and likely unsuccessful, battle, the Collaborative instead decided to implement a two-tiered screening process. Now, any organization in the community can use the screening of their choice to identify older adults at risk for loneliness or social isolation. These individuals are then all referred to the United Way, where the navigators administer U-SIRS as a shared measurement. Three months after the initial intervention by the navigators, U-SIRS is once again administered to measure any changes in isolation risk.

The Navigation subcommittee determined that a team of trained older adult volunteer navigators would administer the intervention, which would consist of individualized phone-based guidance and support to overcome barriers to social connection. The United Way was influential in determining that the navigators could be highly trained volunteers, rather than paid staff. The organization used a similar model successfully to administer its Medicare counseling and tax assistance programs. It was decided that the navigators would receive ongoing education on the ever-changing community resources and organizations that exist to help prevent or reduce an individual's level of isolation. Additionally, priority was placed on identifying navigators in the same age cohort of those residents likely to be served to facilitate trust and understanding when speaking about sensitive topics.

Since implementation, the volunteer older adult navigators have also played a central role in the ongoing administration of the program. They meet as a group regularly with the Program Manager to ensure they have an opportunity to provide feedback on the program, as well as support each other. One of the key input elements they shared is a desire to strengthen their connections with the health and social service agencies they were referring clients to. As a result, the Collaborative implemented monthly lunches, at which the volunteers are hosted by one of the agencies that are part of the Collaborative, to learn more about their services and meet their staff in person.

Finally, recognizing that stigma and lack of awareness about the detrimental health impacts of isolation could represent barriers to individuals accessing this program, several focus groups of local older adults contributed to the ultimate design of an awareness campaign that was launched later in the pilot (Summer 2024). This delay was intentional in order to allow the Collaborative time to refine program operations. The campaign utilizes a combination of media outreach to raise awareness of social isolation and the availability of the navigators as a resource for promoting connection. All three components of the program have been operating from 2022 to 2024 as a 3-year pilot to test the system and will soon transition to full implementation.

### 4.3 Preliminary data

#### 4.3.1 Collaboration

In 2021, The Institute administered an assessment of the Collaborative's internal processes utilizing the Wilder Collaboration Factors Inventory. The Inventory assesses the strength of a collaboration based on 20 evidence-based factors of success (26). The assessment was completed by 22 participants, constituting 44.9% of the Collaborative. Table 2 illustrates the factors and statements from the Inventory that directly corroborate the collective impact model (10). Success within each factor is indicated by a majority of respondents (more than 66%) agreeing or strongly agreeing with the Inventory's given statements. The Institute concluded that most Collaborative members surveyed shared the same vision, firmly believed resolution of the isolation challenge was beyond the capacity of a single organization, and that the Collaborative leadership is skilled. Results from the Inventory suggest that the Collaborative has achieved each of the collective impact elements intended.

**Table 2:** Presentation of select results from the Wilder Collaboration Factors Inventory that correspond with elements of collective impact (10, 26).

**Factor from Wilder Inventory**	**Statement from Wilder Inventory**	**Element of collective impact model**	**Positive responses**
Skilled leadership	The people in leadership positions for this collaboration have good skills for working with other people and organizations	Central infrastructure	17/21 (81%)
Appropriate pace of development	This group is currently able to keep up with the work necessary to coordinate all the people, organizations, and activities related to this collaborative project	Central infrastructure Dedicated staff	18/21 (86%)
Appropriate cross-section of members	The people involved in our collaboration represent a cross section of those who have a stake in what we are trying to accomplish	Common goal	19/21 (90%)
Shared vision	The people in this collaborative group are dedicated to the idea that we can make this project work	Common goal	18/21 (86%)
Members share a stake in both process and outcome	Everyone who is a member of our collaborative group wants this project to succeed	Common goal	21/21 (100%)
Open and frequent communication	People in this collaborative communicate openly with one another	Communication	15/21 (71%)
Established informal relationships and communication links	Communication among the people in this collaborative group happens both at formal meetings and in informal ways	Communication	17/21 (81%)

There have also been organizational and systematic changes in the Lackawanna County older adult service sector. Collaborative members have discussed experiencing a higher degree of coordination in the delivery of aging services in general, as well as a heightened understanding of each other's work. New projects led by subsets of the Collaborative seeking to fill gaps in the region's community service continuum of care have been implemented. For example, the Lackawanna County Area Agency on Aging (LCAAA) partnered with the United Way and local Meals on Wheels affiliate to administer a holiday food distribution for the homebound. This project identified multiple older adults who weren't currently receiving Meals on Wheels, but were eligible. These individuals are now being served. Collaborative leaders have also anecdotally reported observing that individual organizations have shifted in terms of their receptivity to change, welcoming innovation and additional learning opportunities. Currently, five organizations that are part of the Collaborative are participating as a cohort in the Listen4Good program, a national initiative to support nonprofits in gathering and responding to beneficiary feedback (27).

#### 4.3.2 Program process data

Between July 2022 and July 2024, the Collaborative received ~500 referrals from partner organizations for older adults seeking a variety of services. Five volunteer older adult navigators initiated 442 phone calls to the referred older adults. Of those phone calls placed, 252 (57%) resulted in a conversation with an older adult regarding their level of social interaction. Of the 252 phone conversations, 69 resulted in the completion of the U-SIRS screening tool. Some older adults did not wish to take the screening, and in these cases, the navigators offered community service connection support more informally. Of the 69 U-SIRS scores generated, the breakdown in risk score was as follows: 2 (3%) low risk, 6 (9%) medium-low risk, 15 (22%) medium risk, 14 (20%) medium-high risk, and 32 (46%) high risk. These results indicate that of those screened, 88% scored at a risk level which recommends intervention to improve socialization levels.

The navigators recognized that some older adults were more amenable to answering an informal four-item questionnaire than the 13-item U-SIRS assessment. The questionnaire sought to determine if the older adult lives alone, feels isolated from others, lacks companionship, and feels like no one really knows them well. Among the 80 older adults who responded to the short questionnaire, 65 (81%) reported living alone, 31 (39%) reported feeling isolated from others often, 27 (34%) reported lacking companionship often, and 16 (20%) reported often feeling like no one really knows them well. This short form questionnaire is useful as an initial pre-screening tool, the results of which can trigger the navigator to initiate the longer U-SIRS assessment, both of which generate useful data on the participant's social isolation risk. Overall, program process data indicate the Collaborative is reaching older adults who are in need of services to reduce their isolation.

## 5 Discussion

### 5.1 Summary

Preliminary data indicate that the desired elements of collective impact were achieved by the Collaborative. Specifically, the Collaborative developed and has sustained over time the needed organizational infrastructure and programmatic elements (including a well-established and respected community agency sponsor, dedicated leadership, and talented staff and volunteers) for such an initiative. The Collaborative also established a common goal (addressing social isolation among older adults), selected a shared measurement tool (U-SIRS), and fostered open and continuous channels of communication among all stakeholders. Results from the Wilder Collaboration Factors Inventory corresponding with elements of collective impact confirm these accomplishments. A recent systematic review further suggests an intervention by a collaborative network of community stakeholders is a logical and sound approach for enhancing older adult social connection (11). The Collaborative's efforts offer a methodological protocol for how such a model can be achieved and upon completion of further evaluation, is expected to contribute to discourse around the effectiveness of such an approach.

Programmatically, the Collaborative developed a three-component system that screens referred older adults, connects them to trained navigators, and seeks to increase awareness. Preliminary process data have been presented and suggest that the Collaborative is beginning to reach the target audience, older adults experiencing social isolation. Further evaluation will be performed in order to draw conclusions on the impact of the programmatic elements, primarily by assessing changes in baseline and 90 day U-SIRS follow-up scores. Of note is that change in the U-SIRS score has previously been used to measure the impact of a Meals on Wheels America social connection pilot in 2023. That report did note challenges in engaging participants to complete U-SIRS both initially and at follow-up due to language barriers, stigma, and unanswered communications via telephone. The Collaborative has already noted experiencing similar challenges and is incorporating a variety of strategies to assist in overcoming them which they anticipate will be shared when further evaluation has been completed (28).

### 5.2 Implications and lessons learned

Although the pilot program is still in progress, the Collaborative members have discussed several conditions they believe are pivotal for achieving a cohesive and sustainable collective impact initiative. Central infrastructure elements have been particularly key in their feedback (10). For example, the Collaborative recognized the value of an impartial subject matter expert consultant as an external project advisor. The initial idea behind this support was for the expert to provide program design input and keep the group abreast of the developing body of research and best practices related to addressing older adult isolation. While this was accomplished, the selected subject matter expert's contribution was also the only voice in the group without a direct stake in the community and not concerned about representing the interests of a local organization. As a result, the consultant was able to be a crucial, impartial voice of guidance when collaborative decision making proved difficult.

The Collaborative members believe selecting the United Way as the lead organization was also key. The United Way is seen as a “neutral” organization within the community, actively eliminating questions of project ownership and allowing a wide variety of community partners to jointly buy-in to the initiative. Finally, buy-in from the Lackawanna County Area Agency on Aging (LCAAA) is noted as another important element. LCAAA supported the project from the beginning, bringing additional partners to the table, as well as serving as an influential and legitimatizing force and respected clearinghouse of knowledge in terms of how to best serve older adults.

The Collaborative would advise that other communities interested in undertaking similar work consider incorporating a well-respected external perspective, a neutral lead organization, and ensuring buy-in from perceived community-wide leaders in the respective region served.

Beyond central infrastructure components, other aspects of this project that the Collaborative believes played a role in cohesive collective impact were training in the Reframing Aging principles which created a bonding/shared learning opportunity and language; administering the Wilder Collaboration Factors Inventory to identify areas for improvement in the collaborative process; and the development of formal “role description” documents distributed and signed by each member of the collaborative to clarify expectations.

## 6 Challenges

While this collective impact collaboration has been met with strong enthusiasm locally, there are several limitations that must be acknowledged. First, this project still resides in its pilot stage, and, as such, has not yet been fully evaluated to determine effectiveness. A relatively small number of older adults have been served thus far. The third primary component of the pilot, the awareness and education campaign, was launched in 2024, and is still gaining traction in terms of generating referrals from the broader community. It is expected that throughout 2025, as the program transitions from pilot to full implementation, the number of older adults participating will increase significantly, allowing for more in-depth analysis of accumulating data that will help determine the extent of impact of the implemented program design on older adult isolation.

Another limitation is the lack of diversity of volunteers, participants, and Collaborative members in terms of identified race, ethnicity, and cultural background. In response, the Collaborative recently developed a diversity, inclusivity, and equity subcommittee, which has begun seeking input from community diversity experts on how to improve program inclusivity.

The third limitation is the long-term financial sustainability of a collective impact collaboration. Since only the planning and pilot stages have been funded to this point by a group of foundations (Moses Taylor Foundation, The Harry and Jeanette Weinberg Foundation, Northeastern Pennsylvania Health Care Foundation, and Scranton Area Community Foundation), long-term sustainability funding must now be secured to ensure program continuity. Some potential options have been identified and are being pursued for federal and state funding, as well as additional philanthropic support. Furthermore, as a largely rural region, the community has a tradition of resourcefulness as it has considerable experience functioning within ongoing financial constraints. This, too, makes continued commitment to a large-scale collaborative approach even more essential.

## 7 Next steps

The work of the Collaborative will continue for the foreseeable future and multiple next steps are planned or already underway. First, is an expansion of the roster of referral sources across multiple, additional sectors for greater reach and inclusivity. This will be accomplished through individualized outreach to potential partner organizations, as well as the ongoing public awareness campaign.

Second, an outcome and impact evaluation of the Collaborative is planned for 2025. The aims of the program evaluation are to quantify the Collaborative's effectiveness in mitigating social isolation among older adults in Lackawanna County. The evaluation will collect primary data from participants, program staff, and Collaborative members entailing the conduct of an explanatory mixed methods analysis. After the impact evaluation is complete, the Collaborative plans to conduct a multiple-mini case study examining the degree to which the values, norms, and cultural traditions of rural older adults represent a set of potential barriers to care that can be classified as influencing service acceptability. The rural nature of many of the communities in Northeastern Pennsylvania, like elsewhere in rural America, is accompanied by a long-standing tradition of stoicism and a fiercely independent spirit, further impeding many older residents' willingness to seek help and support even when they are aware of existing services (29). The Collaborative members have all anecdotally reported experiencing this rural mindset. Further investigation is needed, though, to describe the extent of this resistance in order to help and identify potential solutions.

Lastly, the Collaborative plans the development of a central referral platform utilizing a mobile application technology to digitally connect the Collaborative members with the public. The long-term goal of the Collaborative is to utilize this central referral platform to maximize low barrier older adult access and to synchronize program communications, referrals, and data collection for future analysis.

## Data Availability

The raw data supporting the conclusions of this article will be made available by the authors, without undue reservation.
